# SARS-CoV-2 IgG Levels as Predictors of XBB Variant Neutralization, Israel, 2022­ and 2023 

**DOI:** 10.3201/eid3005.231739

**Published:** 2024-05

**Authors:** Yaniv Lustig, Michal Canetti, Victoria Indenbaum, Yovel Peretz, Yael Weiss-Ottolenghi, Ili Margalit, Keren Asraf, Tal Levin, Neta Zuckerman, Enosh Tomer, Michal Mandelboim, Ram Doolman, Noam Barda, Gili Regev-Yochay

**Affiliations:** Sheba Pandemic Research Institute, Ramat-Gan, Israel (Y. Lustig, M. Canetti, Y. Peretz, Y. Weiss-Ottolenghi, I. Margalit, N. Zuckerman, G. Regev-Yochay);; Tel-Aviv University Faculty of Medical and Health Sciences, Tel Aviv, Israel (Y. Lustig, M. Canetti, I. Margalit, M. Mandelboim, G. Regev-Yochay);; Central Virology Laboratory, Public Health Services, Ministry of Health, Ramat-Gan (Y. Lustig, V. Indenbaum, T. Levin, N. Zuckerman, E. Tomer, M. Mandelboim);; The Dworman Automated-Mega Laboratory, Ramat-Gan (K. Asraf, R. Doolman);; ARC Innovation Center, Ramat-Gan (N. Barda);; Ben-Gurion University of the Negev, Be’er Sheva, Israel (N. Barda)

**Keywords:** SARS-CoV-2, XBB variant, IgG, antibodies, neutralizing antibodies, viruses, vaccines, COVID-19, vaccine-preventable diseases, respiratory infections, Israel

## Abstract

Although a vaccine against SARS-CoV-2 Omicron-XBB.1.5 variant is available worldwide and recent infection is protective, the lack of recorded infection data highlights the need to assess variant-specific antibody neutralization levels. We analyzed IgG levels against receptor-binding domain–specific SARS-CoV-2 ancestral strain as a correlate for high neutralizing titers against XBB variants.

Since the beginning of 2023, SARS-CoV-2 Omicron XBB variants have led as the cause of global SARS-CoV-2 infections (*1*,*2*). SARS-CoV-2 mRNA vaccines based on the ancestral variant were shown to be less effective against Omicron variants, with reduced neutralization efficiency (*3*,*4*). Because of this reduced neutralization efficiency, updated mRNA vaccines, like the monovalent XBB1.15 vaccine, were developed and distributed (*5*). High levels of neutralizing and receptor-binding domain (RBD) binding IgG levels are known to be correlated with protection from infection or severe disease (*6*,*7*). The evasiveness of Omicron variants against neutralizing antibodies induced by vaccination or infection with previous variants demonstrated the importance of determining variant-specific neutralizing antibodies (*4*). In this study, we investigated the utility of measuring RBD IgG levels against the SARS-CoV-2 ancestral (wild-type [WT]) strain to predict titers of XBB-specific neutralizing antibodies.

During February 2022–August 2023, we obtained 1,070 samples from 373 study participants at Sheba Medical Center in Ramat Gan, Israel, and tested the samples for levels of IgG against WT-RBD and XBB-specific neutralizing antibody levels (Appendix). Most of the study participants were vaccinated >3 times with the BNT162b2 (Pfizer-BioNTech, https://www.pfizer.com) or mRNA1273 (Moderna, https://www.modernatx.com) vaccines, and 39% were previously infected (Table; Appendix Table). Because XBB variants were only marginally circulating in Israel during 2022 but were the dominant variants during 2023 (Appendix Figure 1), we examined antibody levels separately for 2022 and 2023. Although IgG levels against WT virus were lower in 2023 (geometric mean titer of 3,474 binding antibody units [BAU] [95% CI 3,093–3,902] in 2022 vs. 3,971 BAU [95% CI 3,496–4,511] in 2023), 50% inhibitory dilution neutralizing antibody titers against XBB were significantly higher (geometric mean titer of 88 [95% CI 75–1,040] in 2022 vs. 143 [95% CI 121–168] in 2023) 2 (Figure, panel A).

**Table:** Sex, age range, and COVID-19 history of patient participants who provided samples for testing IgG against SARS-CoV-2 ancestral strain and Omicron XBB-specific neutralizing antibody levels in 2022 and 2023, Israel*

Variable	Value
Sex	
F	251 (67)
M	122 (33)
No. COVID-19 vaccinations received	
0	1 (0.3)
1	13 (3.5)
2	5 (1.3)
3	102 (27)
4	215 (58)
5	36 (9.7)
6	1 (0.3)
No. COVID-19 infections	
0	227 (61)
1	120 (32)
2	22 (5.9)
3	3 (0.8)
4	1 (0.3)

*Values are no. (%) except as indicated.

**Figure:**
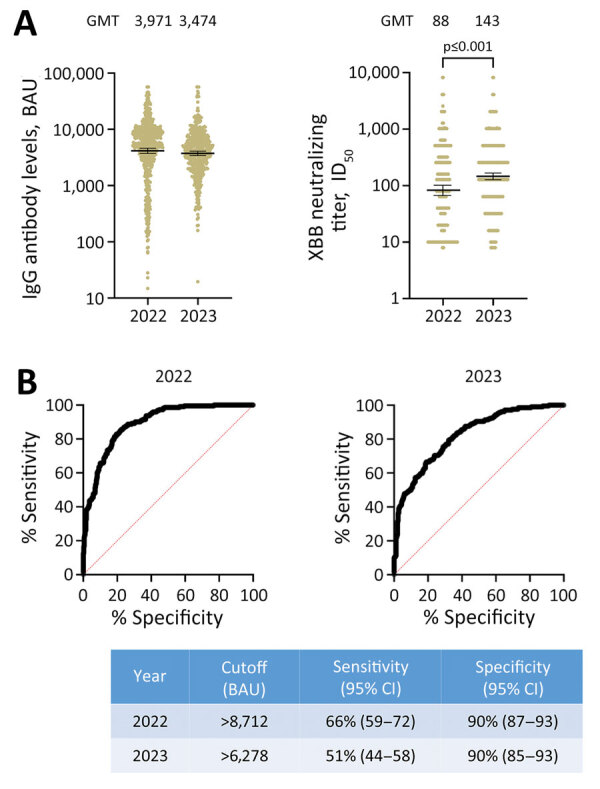
Binding IgG and neutralizing titer levels from samples collected in 2022 and 2023 from patient participants at the Sheba Medical Center, Israel, and the prediction of SARS-CoV-2 Omicron XBB neutralization by RBD-WT IgG levels from those samples. A) Scatter plot analyses of 1,071 WT IgG and XBB-specific neutralizing titers in samples obtained from healthcare workers during 2022 and 2023. Horizontal lines indicate GMTs; error bars indicate 95% CIs. GMT of each timepoint is indicated. B) ROC curves showing the diagnostic value of WT IgG levels for high (titer >250) XBB-specific neutralization levels. Sensitivity and specificity determinants for specific cut off levels are shown. BAU, binding antibody unit; GMT, geometric mean titer; ID_50_, 50% inhibitory dilution; RBD, receptor-binding domain; ROC, receiver operating characteristic; WT, SARS-CoV-2 ancestral (wild-type) strain.

We assessed the correlation between WT IgG and XBB neutralizing antibody levels. Although a strong correlation between RBD IgG and neutralizing antibody titers was maintained in both years, a stronger correlation was detected in 2022 (repeated measures correlation of 0.54 [95% CI 0.46–0.60]) compared with 2023 (repeated measures correlation of 0.31 [95% CI 0.17–0.44]). The regression co-efficient between IgG and neutralizing antibody levels was different for 2022 and 2023 (Appendix Figure 2). We found the expected value of XBB specific neutralizing antibody titers for IgG of 7,000 BAU was 156 in 2022 and 276 in 2023.

We investigated if the correlation between WT IgG and XBB neutralization levels could be applied to predict persons with high XBB neutralization titers. A titer of 50% inhibitory dilution >1:250 was considered to be high neutralizing. US Food and Drug Administration guidelines consider titers of 50% inhibitory dilution >1:250 as eligible for transfusion as COVID-19 convalescent plasma (*8*,*9*). We found 36% of samples in 2022 and 46% of samples in 2023 had 50% inhibitory dilution >1:250. The area under the receiver operating characteristic curve was 0.89 (95% CI 0.87–0.92) for 2022 and 0.82 (95% CI 0.79–0.86) for 2023, suggesting a good discrimination between high and low titers based on WT IgG levels. Requiring a specificity of 90%, the receiver operating characteristic analysis showed a sensitivity of 66% (95% CI 59%–72%) for WT IgG levels >8,712 BAU in 2022 and a sensitivity of 51% (95% CI 44%–58%) for WT IgG levels >6,278 BAU in 2023 (Figure, panel B).

The results of our study show that measuring IgG against the SARS-CoV-2 ancestral strain (WT-RDB) can predict the presence of high neutralizing antibody levels against current circulating variants. We focused on the prediction of neutralizing antibodies against XBB variants because it was the immune antigen present in the vaccines available during the study period. We found that significantly higher XBB neutralizing antibody titers, but lower WT-RBD IgG levels were detected in samples obtained during 2023 compared with 2022. One explanation is that increased exposure to XBB-related variants in 2023 led to the development of XBB-specific antibodies paired with waning WT IgG levels. Our regression co-efficient analysis showed that samples obtained in 2022 had higher mean WT IgG levels than in 2023, despite having similar XBB neutralizing levels. The WT IgG level cutoff that can predict XBB-specific high neutralizing antibodies with 90% specificity was lower in 2023 compared with 2022.

The continued waves of COVID-19 infections together with SARS-CoV-2 vaccinations have diversified the immune protection of humans worldwide. Vital public health actions to prevent COVID-19 infections include prioritizing vaccination on the basis of known immunity, estimating the immune status of the population, ensuring COVID-19 convalescent plasma has high neutralizing antibodies, and investigating the effects of updated vaccines in persons with varying levels of neutralizing antibodies. Our results show that, regardless of any knowledge of previous SARS-CoV-2 infections, WT IgG levels are correlated and can predict XBB-specific neutralizing antibody titers.

Appendix Additional information about SARS-CoV-2 IgG levels as predictors of XBB variant neutralization, Israel, 2022 and 2023.
