# Supplementary material for: SARS-CoV-2 IgG Levels as Predictors of XBB Variant Neutralization, Israel, 2022­ and 2023
Source: Emerg Infect Dis. 2024 May;30(5):1050–2. doi: 10.3201/eid3005.231739 (PMC11060444; doi:10.3201/eid3005.231739)
Supplement: Appendix — Additional information about SARS-CoV-2 IgG levels as predictors of XBB variant neutralization, Israel, 2022 and 2023. [file 23-1739-Techapp-s1.pdf]

Article DOI: https://doi.org/10.3201/eid3005.231739

EID cannot ensure accessibility for supplementary materials supplied by authors. Readers who have difficulty accessing supplementary content should contact the authors for assistance.

SARS-CoV-2 IgG Levels as Predictors of XBB Variant Neutralization, 2022 and 2023

Appendix

Materials and Methods

Study Samples

All of the study participants were part of the Sheba COVID-19 study cohorts. These include health care workers (HCW) from the Sheba serology study (*1*,*2*), which was initiated before the rollout of the first COVID-19 vaccine dose and volunteers from clinical studies initiated by Sheba (*3*,*4*). All of Sheba COVID-19 study cohorts participants (SSP) were encouraged to undergo antigen rapid diagnostic testing (Ag-RDT) or quantitative real-time polymerase chain reaction (qRT-PCR) for SARS-CoV-2 detection in the event of exposure to an infected person or if they exhibited any COVID-19–related symptoms. Additionally, SSP were encouraged to test weekly and received reminders through emails, text messages, or phone calls. All cohort members were asked to perform serology testing monthly.

The 1070 samples included in this study were randomly obtained from 373 Sheba Medical Center Study Participants from February 23rd 2022 until August 16th 2023. Since each SSP donated several samples to this study, Table 1 shows the demographic and SARS-CoV-2 exposure (COVID-19 vaccination and infection) history of SSP and appendix Table 1, the SARS-CoV-2 exposure history of each sample.

Statistical Analysis

Geometric Mean Titers (GMTs) with 95% confidence intervals were estimated for each antibody each year by fitting an intercept-only linear regression model with the logarithm of the antibody measurement as the outcome, with a random effect per person to account for repeated

measurements. A direct comparison between years was performed by fitting the same model with the year as the sole predictor.

Correlation between the two antibody types was estimated for each year using repeated measures correlation [https://www.frontiersin.org/articles/10.3389/fpsyg.2017.00456/full]. A linear regression with XBB neutralizing antibodies as the outcome, WT IgG antibodies as the sole predictor, and a random effect per person, was used to estimate and plot a regression line, and to estimate the expected value of XBB antibodies for a person with IgG antibody levels of 7000. A confidence interval for this prediction was estimated using the non-parametric percentile bootstrap with 1000 repetitions.

ROC curves were estimated and plotted in the standard manner, with the sensitivity level at 90% specificity noted. A 95% confidence interval for this level was estimated using the exact binomial distribution.

Serology Assays

Samples were tested using the SARS-CoV-2 IgG II Quant (6S60, Abbott) test. These commercial tests were performed according to the manufacturer's instructions.

A SARS-CoV-2 lentivirus-based neutralization assay was performed to assess XBB-specific (either XBB1.9 or XBB1.16) neutralizing antibody levels measured in 50% inhibitory dilution (ID50). Neutralization assay was adapted from (*5*) with minor modifications.

Lentiviral particles were produced by co-transfecting HEK293T/17 cells with an expression vector encoding variant specific SARS-CoV-2 spike alongside packaging vector pCMVDR8.2, luciferase reporter vector pHR′CMV-Luc and a TMPRSS2 expression vector (a gift from Dr. Daniel Douek, Vaccine Research Institute (VRC), National Institute of Health (NIH). MD, USA). Supernatant was collected from cells 48-hour post transfection and used for subsequent neutralization. Transfection was done using Lipofectamine 3000 (Thermo Scientific, cat# L3000001) as specified by the manufacture.

For neutralization, serum samples were heat inactivated in 56°C for 30 minutes. Serum samples were 2-fold diluted in a 96-well plate in dilution medium (MEM 5% FBS), overlaid with pseud-typed Lentivirus solution and incubated in 37°C for one hour. Pseudovirus-serum complexes were than overlaid with HEK293 TMPRSS2-ACE2 cells suspended in dilution

medium. Cells were incubated in 37°C for 72 hours. Following incubation, luminesces was quantified by lysing the cells with tissue culture lysis reagent (Promega, cat# E1531) and adding luciferase assay substrate (Promega, cat# E1501). Luminescence was read using a Varioskan LUX Multimode Microplate Reader (Thermo Scientific).

References

1. Gilboa M, Gonen T, Barda N, Cohn S, Indenbaum V, Weiss-Ottolenghi Y, et al. Factors associated with protection from SARS-CoV-2 omicron variant infection and disease among vaccinated health care workers in Israel. JAMA Netw Open. 2023;6:e2314757. PubMed https://doi.org/10.1001/jamanetworkopen.2023.14757

2. Lustig Y, Sapir E, Regev-Yochay G, Cohen C, Fluss R, Olmer L, et al. BNT162b2 COVID-19 vaccine and correlates of humoral immune responses and dynamics: a prospective, single-centre, longitudinal cohort study in health-care workers. Lancet Respir Med. 2021;9:999–1009. PubMed https://doi.org/10.1016/S2213-2600(21)00220-4

3. Barda N, Lustig Y, Indenbaum V, Zibly D, Joseph G, Asraf K, et al. Immunogenicity of Omicron BA.1-adapted BNT162b2 vaccines: randomized trial, 3-month follow-up. Clin Microbiol Infect. 2023;29:918–923.e7. PubMed https://doi.org/10.1016/j.cmi.2023.03.007

4. Canetti M, Barda N, Gilboa M, Indenbaum V, Mandelboim M, Gonen T, et al. Immunogenicity and efficacy of fourth BNT162b2 and mRNA1273 COVID-19 vaccine doses; three months follow-up. Nat Commun. 2022;13:7711. PubMed https://doi.org/10.1038/s41467-022-35480-2

5. Pegu A, O'Connell SE, Schmidt SD, O'Dell S, Talana CA, Lai L, et al. Durability of mRNA-1273 vaccine-induced antibodies against SARS-CoV-2 variants. Science. 2021;373:1372–7. PubMed https://doi.org/10.1126/science.abj4176

Appendix Table. Previous exposure of the samples used in this study in 2022 and 2023.

Variable	*2022, N = 650*[1]	*2023, N = 420*[1]
Number of COVID-19 Vaccines		
0	0 (0%)	2 (0.5%)
1	20 (3.1%)	18 (4.3%)
2	3 (0.5%)	4 (1%)
3	38 (5.8%)	112 (27%)
4	520 (80%)	250 (60%)
5	69 (11%)	33 (7.9%)
6	0 (0%)	1 (0.2%)
Number of COVID-19 Events Documented		
0	519 (80%)	250 (60%)
1	111 (17%)	138 (33%)
2	19 (2.9%)	28 (6.7%)
3	1 (0.2%)	3 (0.7%)
4	0 (0%)	1 (0.2%)

[1]n(%)

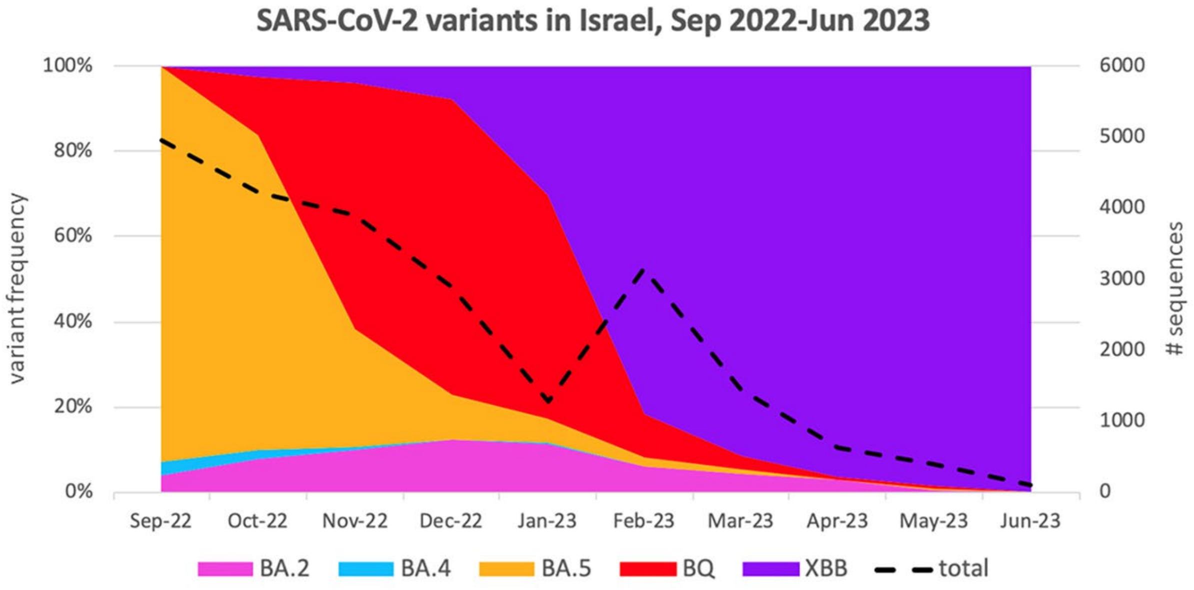

Appendix Figure 1. SARS-CoV-2 variants circulating in Israel between September 2022 and June 2023. Variant frequency of sequenced SARS-CoV-2 positive samples in Israel, depicted over time (left y-axis). The dashed line (right y-axis) represents the number of total sequenced SARS-CoV-2 positive samples over time.

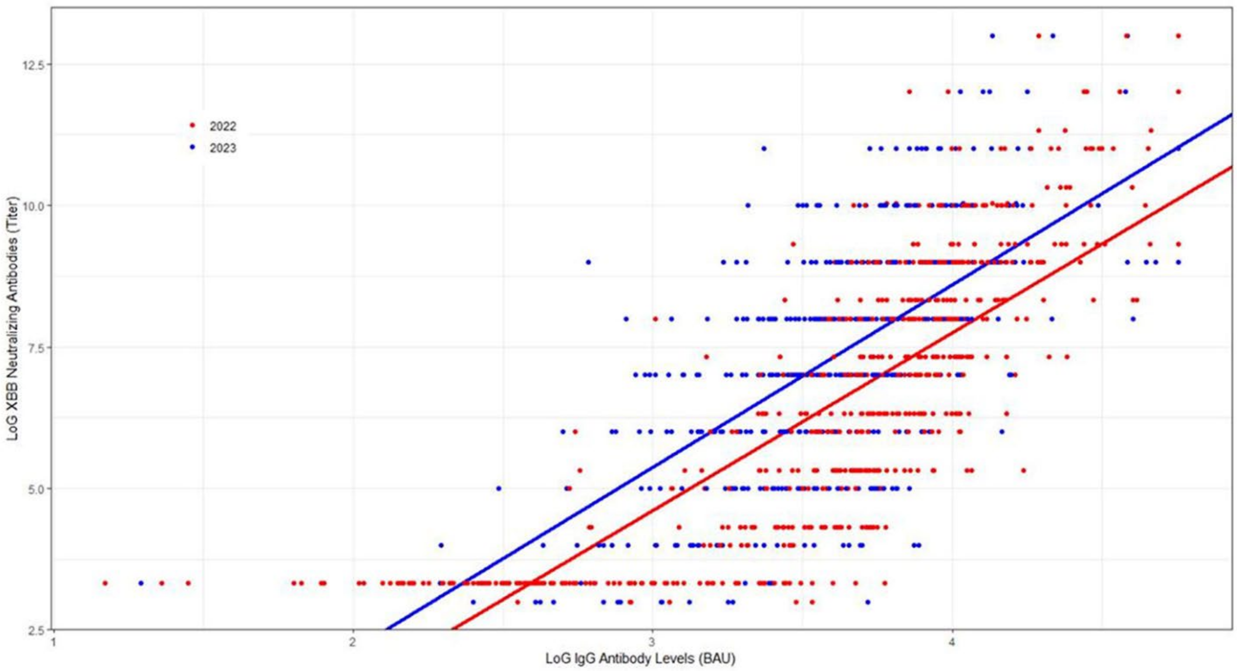

Appendix Figure 2. Correlation of XBB neutralization by RBD-WT IgG antibody levels. Correlation of anti RBD IgG antibody levels with XBB specific neutralizing titers in 2022 (red) and 2023 (blue). Each line was obtained by linear regression and represents the association between binding and neutralizing antibodies in 2022 and 2023.
